# 25th Anniversary of Molecules—Recent Advances in Inorganic Chemistry

**DOI:** 10.3390/molecules26092589

**Published:** 2021-04-29

**Authors:** Burgert Blom, Erika Ferrari, Vassilis Tangoulis, Cédric R. Mayer, Axel Klein, Constantinos C. Stoumpos

**Affiliations:** 1 Maastricht Science Programme, Assistant Professor of Inorganic Chemistry and Catalysis, Maastricht University, Kapoenstraat 2, P.O. Box 616, 6200 MD Maastricht, The Netherlands; 2 Department of Chemical and Geological Sciences, University of Modena and Reggio Emilia, Via Campi 103, 41125 Modena, Italy; erika.ferrari@unimore.it; 3 Department of Chemistry, University of Patras, 26504 Patras, Greece; vtango@upatras.gr; 4 Laboratoire LuMin, FRE CNRS 2036, CNRS, Université Paris-Sud, ENS Paris-Saclay, Centrale Supelec, Université Paris-Saclay, F-91405 Orsay CEDEX, France; cedric.mayer@uvsq.fr; 5 Department für Chemie, Institut für Anorganische Chemie, Universität zu Köln, Greinstraße 6, 50939 Köln, Germany; axel.klein@uni-koeln.de; 6 Department of Chemistry, Northwestern University, Evanston, IL 60208, USA; konstantinos.stoumpos@northwestern.edu

Celebrating the “25th Anniversary of Molecules” with a Special Issue dedicated to “Recent Advances in Inorganic Chemistry” strengthens the renewed role that inorganic chemistry, one of the oldest chemistry divisions, has lately earned thanks to cutting-edge perspectives and interdisciplinary applications, eventually receiving the veneration and respect which its age might require [1,2].

The last 25 years have seen staggering advances in both solid-state, molecular, catalytic and bio-inorganic chemistry. Some notable highlights in molecular inorganic chemistry over the last 2.5 decades certainly must include the propensity of heavy p-block elements (those in period 3 or higher) to undergo multiple bonding with other heavy p-block elements (double and triple bonds) or form multiple bonds with transition metals. This is a field that is technically very demanding given the high reactivity of the emerging systems, which require kinetic protection. Indeed, work that has been feverishly conducted in this area, has demonstrated that multiple bonding between p-block elements opens new vistas into novel reactions over these unsaturated bonds and combinations of heavy p-block elements with transition metals has enabled fascinating new types of catalysts for a variety of synthetic transformations. Hence, it can readily be said that the last 25 years has seen a rebirth of molecular main-group organometallic chemistry combined with more traditional transition metal-based organometallic chemistry and a lot of work has been conducted at this interface. These pursuits are likely to continue into the future given the exciting new chemistry that has been uncovered in the last two decades.

Another area that has certainly witnessed dramatic advances in this time period is the field of biomimetic catalysis and metal-based drug discovery. Building well-defined models to gain insights into enzymes in nature has kept the attention of many researchers world-wide, and perhaps a good example of this is the advances in models of different hydrogenases. Excellent progress has been made in this direction, in particular understanding the reactive core of naturally occurring enzymes by using the tool kit available for inorganic synthetic chemists. This, along with dramatic advances in spectroscopic techniques such as EPR, NMR, magnetometry, and mass spectrometry, facilitated by increased computing power and engineering developments, has enabled a much more detailed understanding of such systems, hitherto inaccessible. Advances in computational methods—in particular, density functional theory (DFT)—which have undergone massive deployment and growth in the last 25 years, has enabled facile entry to understand the electronic and bonding situation of complex biomimetic and other systems. The use of DFT as a support of synthetic and spectroscopic work in inorganic chemistry has generally come into focus, has facilitated increased collaboration between synthetic, spectroscopic and theoretical inorganic chemists and has become routine in the last 25 years.

The synthesis of metal-based drugs has also come into sharp focus in the last 25 years, and substantial progress has been made (for example) in the synthesis and testing of a plethora of transition metal based anti-cancer drugs. Not only have thousands of new, well-defined metal-based systems been discovered that are biochemically active, but detailed mechanistic studies have also been reported, shedding light on novel metal-based drug systems for use in therapy. This has not only been restricted to cancer, but also many other fields, such as anti-microbial studies. This area of investigation is likely to continue unabated into the future given the advances made recently. Finally, a staggering array of new metal-based catalysts have been discovered over the last 25 years and new metal-based catalytic transformations have been uncovered. A strong theme has been the inspiration from nature and again, understanding enzymes in nature has served as a “leitmotif” for the rational design of new metal-based homogeneous catalyst systems. Finally, in the field of solid-state chemistry, a great many new materials have been reported featuring interesting magnetic, thermal, tensile, electrical and chemical properties. These materials open the door to exciting new materials for applications in medicine, engineering, optics, and as new semi-conductors in electronics. These are just some highlights in what is a very broad and rapidly developing field. The general development of the field in the last 25 years has been one of moving towards more collaboration, interdisciplinary investigations, and applications-based research, while at the same time pursuing fundamental “blue-sky” molecular and solid-state inorganic chemistry.

In this evolving landscape, it was an honour to be Guest Editors to this Special Issue that attempts to keep up with the recent developments in inorganic chemistry research and collects twelve manuscripts, including one communication, eight original papers and three reviews. The submitted articles show top-quality level innovations and provide the reader with an overview of contemporary inorganic chemistry, in which coordination chemistry serves as the basis for the development of smart inorganic materials for several applications.

Inorganic chemistry is becoming more and more interdisciplinary, providing innovative uses and approaches for ancient metal elements that can find new life in biomedical applications. Currently, the antimicrobial and antifungal activity of silver is exploited in a very miscellaneous set of applications ranging from simple consumer goods to complex medical devices. Among all, cutting-edge approaches are represented by the use of polyoxometalate (POM) cluster [3] and silver-based coordination polymers [4]. Another interesting tactic in the development of new therapeutics is the combination of inorganic nanoparticles with biomimetic nanocarriers, such as exosomes, that has recently paved the way to novel approaches in bioimaging and therapy [5]. On the other hand, despite its important role in biological systems and pharmaceutical applications, copper(I) can be exploited to manufacture organic light-emitting diodes (OLEDs) and light-emitting electrochemical cells (LECs), as highlighted by Meyer et al., particularly as heteroleptic complexes [6]. Similarly, iron-based ferrocenylsubphthalocyanines have been recently used to design dyads due to their tunable redox behaviour [7], while magnetite nanoparticles, when surface-functionalised with organophosphonates, allow the reversible formation and cleavage of organic bonds with high potential in switchable applications such as self-healing materials [8]. Hierarchical structures composed of materials, also called hybrid materials, may address specific environmental issue such as wastewater treatment; da Silva et al. report the development of Fe alloys and Fe-oxides dispersed in mesoporous matrixes that provide new nanodispersed materials able to adsorb organic molecules (mainly dyes) present in wastewater followed by magnetic separation [9]. Most catalysts are based on inorganic compounds, and among them, *d*-block metals have shown important applications in heterogeneous catalysis throughout the last decades. The use of supporting materials allows improving both the catalytic properties and the stability under harsh reaction conditions. In this Issue, three innovative and original investigations dealing with vanadium-containing faujasite zeolite [10], TiO_2_ thin-films [11] and Ni(OH)_2_ nanoclusters [12] are described.

In reporting the most recent advances in inorganic chemistry, we did not forget the endohedral metallofullerenes, which were left unexplored until 2016 due to the difficulty of experimentally pursuing these hybrid molecules with equivalent carbon cages to their pristine “empty” forms, as extensively reviewed by Yamada et al. [13].

Finally, we must not forget the other side of the coin, i.e., the toxicity that both endogenous and exogenous metal ions have demonstrated and their role in many disorder-related diseases. Lately, an increasing concern in facing neurological diseases and cognitive impairment has driven research to unravel the role of metals in these diseases’ pathogenesis. The possible associations of the elemental status of Mg, Fe, Zn, Cu and Se with attention-deficit/hyperactivity disorder (ADHD) occurrence is here extensively reviewed by Robberecht et al. [14]. The overview delivers interesting outcomes, such as the possible correlation between metal depletion and the development of ADHD, suggesting the potential role of metal concentration in blood as biomarkers for this disease.
